# Normalization of temperature effects for quality assurance of quantitative prostate apparent diffusion coefficient imaging across multiple sites

**DOI:** 10.1002/acm2.70311

**Published:** 2025-11-27

**Authors:** Ken‐Pin Hwang, Joshua Yung, R. Jason Stafford, Caroline Chung, Aradhana M. Venkatesan

**Affiliations:** ^1^ Department of Imaging Physics The University of Texas M.D. Anderson Cancer Center Houston Texas USA; ^2^ Department of Radiation Oncology The University of Texas M.D. Anderson Cancer Center Houston Texas USA; ^3^ Department of Abdominal Imaging The University of Texas M.D. Anderson Cancer Center Houston Texas USA

**Keywords:** apparent diffusion coefficient, diffusion weighted imaging, prostate imaging, quality assurance, temperature effects on diffusion

## Abstract

**Background:**

Apparent Diffusion Coefficient (ADC) as measured by diffusion weighted imaging is known to negatively correlate with prostate tumor aggressiveness. Heterogeneity in system and protocol performance causes potential variability in ADC acquired across a large scanner network, prompting a need to evaluate quantitative ADC from a prostate‐specific MR diffusion protocol as part of quality assurance (QA). Due to the temperature dependence of ADC, repeatability and reproducibility assessments typically require phantoms to maintain a temperature of 0°C, imposing a considerable burden when assessing large numbers of scanners.

**Purpose:**

To develop a QA procedure at room temperature for assessing the reproducibility of ADC measured by our prostate diffusion MRI protocols, by employing a model relating ADC and phantom gel concentration to temperature.

**Methods:**

A diffusion phantom was imaged with our clinical prostate diffusion protocols on 1.5 T (*n* = 4) and 3 T (*n* = 4) scanners in separate geographical locations (*n* = 4). Phantom temperature was not strictly controlled but measured immediately before and after each acquisition. Regions of interest were drawn in phantom compartments to produce ADC measurements at varying phantom gel concentrations. The ADC temperature model was applied using measured temperatures to produce normalized ADC measurements at a reference temperature of 20°C. Percent difference and coefficient of variation (CV) calculations were performed on the measurements with and without normalization.

**Results:**

Temperature normalization reduced the range of ADC percent difference across scanners. The maximum CV of clinically relevant ADC values was reduced from 5.0% to 3.4% when normalized to the temperature model, while CV of pure water was reduced from 3.3% to 1.3%.

**Conclusion:**

We have developed a QA procedure that incorporates a temperature model to obviate the need for strict temperature control of a diffusion phantom, facilitating ADC reproducibility assessments of a prostate diffusion MRI protocol across a large scanner network.

## 1 INTRODUCTION

Among American men, prostate cancer is the most commonly diagnosed solid organ cancer and the second leading cause of cancer‐related death.^1^ Prostate cancer presents a wide spectrum of risk categories, with accurate risk stratification being essential for optimal clinical management. Multiparametric MRI of the prostate incorporates dynamic contrast‐enhanced and diffusion‐weighted imaging (DWI) techniques that may improve detection and risk stratification. DWI with different diffusion weightings measures the microscopic mobility of water, quantified as the apparent diffusion coefficient (ADC). Since increased cellularity can restrict water diffusion, ADC has become an important biomarker in cancer imaging^2^ and has been negatively correlated with Gleason score, the most common system used to grade aggressiveness of prostate cancer.^3^, ^4^, ^5^ The ability for ADC to distinguish incremental risk categories would be impaired by a lack of precision in its measurement. Among the major causes of ADC imprecision are the disparate scanner vendors, models, and field strengths used by large imaging networks. These necessitate variations in acquisition parameters that can impact ADC quantitation.^6^ For patients scanned serially on different scanners, ADC variability that is a result of scanner variation can confound true differences in ADC that are due to tumor aggressiveness or treatment response.

The Quantitative Imaging Biomarker Alliance (QIBA) has released guidelines regarding variability in quantitative imaging results across imaging platforms, clinical sites, and time. It has published guidelines for the measurement of quantitative biomarkers in terms of their accuracy, repeatability, and reproducibility^7^ and released the QIBA diffusion profile,^8^ which outlines measurement methods and their specifications to qualify sites for participation in multi‐center imaging trials. The profile was developed based on previous work using a compartment of pure distilled water chilled to 0°C in an ice‐water bath to maintain its temperature during scanning.^9^, ^10^, ^11^ The primary disadvantage with this type of phantom is the need to achieve a consistent phantom temperature of 0°C, which can necessitate immersion for 2–3 h with an ice‐water bath to ensure thermal equilibrium throughout the phantom. Despite the challenges of this procedure, it serves well as a universal standard since temperature is known to affect the diffusivity of water by roughly 2%3%,^2^, ^12^, ^13^, ^14^ and any heat absorbed by the ice‐water mixture at 0°C would melt some of the ice instead of raising the temperature of the mixture. While the QIBA profile does provide recommendations for specific scan parameters when imaging certain anatomical sites, including brain, breast, and prostate, the scan parameters for the protocol used to characterize scanners are primarily tailored to brain imaging protocols. Expanding on this phantom design, the National Institute of Standards and Technology (NIST) has collaborated with QIBA and the Radiological Society of North America (RSNA) to design an isotropic diffusion phantom^15^, ^16^ consisting of multiple compartments containing solutions of varying concentrations of the polymer polyvinylpyrrolidone (PVP), which can increase the ADC values of the solutions to be comparable to those encountered in human tissues.^17^ Phantoms of this design have been evaluated in recent studies to characterize repeatability and reproducibility of diffusion measurements.^18^, ^19^, ^20^, ^21^

Our institution has a large network of over 30 scanners situated at four separate geographical locations, and multiparametric prostate MR exams are performed on all of them. These scanners are a mix of two vendors and two field strengths, with multiple models and/or software versions for any given combination of vendor and field strength. While most prostate exams are performed with an endorectal coil, our 3 T scanners are also configured with a protocol for scanning without an endorectal coil. Given the wide variety of scanners, large number of scanning protocols, and large volume of prostate exams, scheduling patient exams on specific scanners is impractical due to the impact it can have on overall clinical workflow. As a result, patients are typically imaged on several different scanners over the course of treatment, introducing potential variability to the quantitative ADC measurements in the multiparametric exam. Thus, there exists a need for a quality assurance (QA) process that can characterize the variability of this measurement. Ideally, this process would be simple to execute on multiple scanners sited at different locations while also being specific to dedicated prostate multiparametric MR protocols. Compared to phantoms maintained at 0°C, phantoms at room temperature are more convenient to prepare,^22^, ^23^, ^24^ but require a correction to account for the temperature dependence of ADC.^22^ Recently, models for this temperature dependence have been developed for the PVP solutions used in the NIST phantom.^25^, ^26^ New measurements by NIST at a range of temperatures near room temperature were also recently released, which potentially may serve as reference values.^27^ In this study, we investigate the feasibility of this model as a correction factor for ADC measurements acquired at room temperature for multiparametric prostate protocols imaged across multiple MR scanners within our institutional network.

## 2 MATERIALS AND METHODS

The NIST diffusion phantom (Diffusion Phantom, CaliberMRI, Boulder, Colorado, United States) is a commercially available phantom consisting of 13 vials: a center vial, surrounded by a ring of 6 vials, which are surrounded again by a ring of 6 vials. The vials contain Polyvinylpyrrolidone (PVP) in aqueous solution in concentrations of 0%, 10%, 20%, 30%, 40%, and 50%. The phantom was scanned on four 1.5 T scanners (MAGNETOM Aera, Siemens Healthineers, Erlangen, Germany) and four 3 T scanners (three MAGNETOM Skyra and one MAGNETOM Vida, Siemens Healthineers, Erlangen, Germany), at four separate geographical sites across our institutional network. Phantom temperatures were measured using a long stem thermometer (Traceable, Webster, Texas, United States) by positioning as much of probe tip as possible in the main water compartment. At each site, the water in the main compartment of the phantom was prepared for scanning by adjusting with warm water or ice to match the air temperature measured in the room to within 0.5°C, making sure that any added ice had completely melted and water was well mixed within the phantom when the temperature was measured. Alternatively, the phantom could be left in the room to equilibrate over several hours, but such a process was not utilized for this study. The temperature of the phantom water was measured a second time immediately after scanning was completed.

The phantom was positioned for scanning at a designated location on the scan table equipped with the spine array coil and stabilized with pads surrounding the base of the phantom; no other external coils were utilized. The phantom was imaged with the small FOV Single Shot Echo Planar Imaging DWI sequence employed for our multiparametric prostate MR protocol, adapted for use with the diffusion phantom by allowing two main modifications: utilization of only the spine coil without the endorectal or torso array coil, and imaging in the coronal plane instead of an oblique axial plane. Since our non‐endorectal coil protocol is only run on 3 T, both endorectal and non‐endorectal versions of our prostate protocol were tested at 3 T. Scan parameters are shown in Table 1.

**TABLE 1:** Scan parameters for the three prostate DWI protocols evaluated in this study.

	1.5 T Aera	3 T (Skyra, Vida), endorectal	3 T (Skyra, Vida), non‐endorectal
TR	5400–5700	5400, 5200	4600, 4800
TE	55	56 (Skyra), 51 (Vida)	59–61 (Skyra), 52 (Vida)
FOV	180	180	200
Matrix	96 × 80	96 × 80	80 × 80
Phase oversampling	50%	50%	50%
Partial Fourier	6/8	6/8	7/8
Slice thickness	3	3	3
Slice spacing	0	0	0
Number of slices	30	30	30
Diffusion mode	3‐scan trace	3‐scan trace	3‐scan trace
*b*‐values	50, 800	50, 800	50, 800
NEX	2, 16	2, 16	2, 16
Bandwidth/pixel	1860	1580	2016
Interpolation	On	On	On
GRAPPA acceleration	2	2	2
Fat suppression	SPAIR	SPAIR	SPAIR

ADC values were measured within each of the vials of the phantom by placing circular regions of interest (ROI's) of 1.5 cm diameter on a slice exhibiting minimal distortion and artifacts. Since the diameter of the ROI's was smaller than the diameter of the vials, the exact placement of each ROI was adjusted to minimize overlap with ringing or distortion artifacts of the vial walls. The mean of the pixel values within the ROI was taken as the ADC measurement for that vial. Altogether, for a given protocol, data were collected for 13 vials and 12 scanner‐protocol combinations: four 1.5 T scanners using the endorectal protocol, four 3 T scanners using the endorectal protocol, and the same four 3 T scanners using the non‐endorectal protocol. The well‐established Speedy‐Angell model for temperature dependence of ADC of pure water^28^, ^29^ was used with a quadratic calibration function for PVP vial concentration^26^ to predict ADC values based on measured temperature in our experiments. The temperature model for pure water is governed by the equation

$$\;ADC_{W}=exp\left(c_{1}+c_{2}\cdot \left(1000/T\right)\right)$$

where ADC_W_ is the ADC of water, T is the temperature of the water, and c_1_ and c_2_ are coefficients provided Speedy‐Angell model (typically 8.09 and –2.17) that describe the temperature dependence of ADC. The calibration for PVP concentration scales ADC of a PVP solution relative to ADC of pure water and is given by a quadratic function fitted by Amouzandeh et al:

$$\;\frac{ADC_{PVP}}{ADC_{W}}=K1\cdot {\left[PVP\right]}^{2}+K2\cdot \left[PVP\right]+1$$

Here, ADC_PVP_ is the ADC of the PVP solution, ADC_W_ is the ADC of pure water, and K1 and K2 are fitted parameters to the equation. Another set of reference ADC values was based on measurements taken by NIST for their new diffusion phantom.^27^ Those measurements were acquired at temperatures ranging from 16°C to 26°C in discrete increments of 2°C. For this study, these discrete ADC values were interpolated based on the measured phantom temperatures to provide more precise ADC values at temperatures between the discrete temperatures.

In our analysis, the temperature of the phantom during scanning in the scanner was defined as the average of the temperatures measured before and after scanning.^24^ The temperature model was used to produce two sets of reference ADC values for each vial and scanner: one assuming a fixed room temperature of 20°C (ADCs_20C_) and another using the temperature of the phantom while in the scanner (ADCs_Temp_). A third set of reference ADC values was taken as the values measured by NIST, interpolated to the temperature of the phantom measured while in the scanner (ADCs_NIST_). Bias calculations were not possible since no gold standard ADC values were provided for this iteration of the phantom at room temperatures. Rather, vial measurements were normalized to each of the three sets of reference ADC values to produce three percent difference calculations:

$$\;\%Diff_{ROI}=\left(\frac{ADC_{ROI}-ADC_{REF}}{ADC_{REF}}\right)\;\times 100\%$$

Here, ADC_ROI_ is the mean of the values within an ROI, ADC_REF_ is the reference diffusion coefficient (ADC_Temp_, ADC_20C_, or ADC_NIST_). For each vial and reference value, the coefficient of variation (CV) across scanners and protocols was calculated as a measure of intra‐scanner reproducibility with the following equation:

$$CV=\frac{\sigma }{\mu }$$

For a given vial, σ is the standard deviation of the mean ROI ADC measurements across scanners and protocols, and μ is their mean. CVs were calculated across all scanners and protocols, for 1.5 T scanners, 3 T scanners, and all scanners combined. Statistical significance of differences between reference values was evaluated with Levene's test for equality of variances.^30^, ^31^

## 3 RESULTS

**FIGURE 1:**
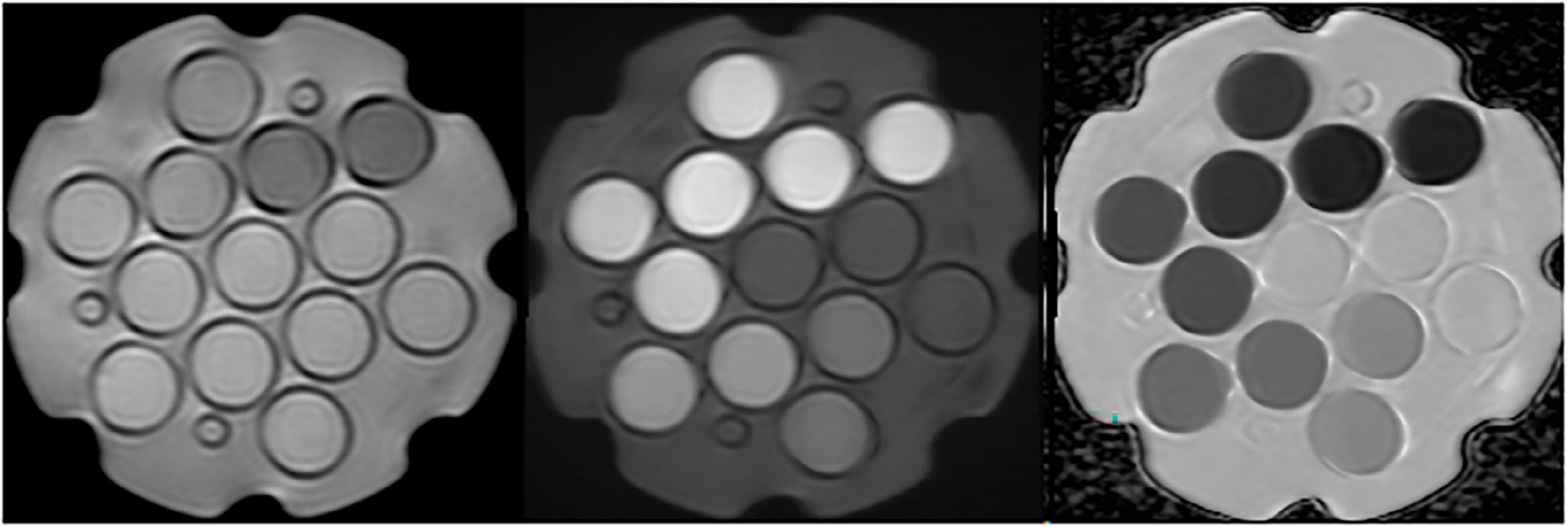
Trace b = 50 (left), trace b = 800 (center), and ADC (right) images of the NIST phantom, which contains 13 vials of varying concentrations of PVP: three at 0%, and two each at 10%, 20%, 30%, 40%, and 50%.

Representative trace and ADC images are presented in Figure 1. Since minimal distortion was observed in slices near the center of the vial below the central plate, measurements were taken in this area. While data for 7 of the 8 scanners were measured within a span of 83 days, the Vida 3 T scanner was being installed during these initial assessments and could not be evaluated until 175 days after the first measurement. Mean phantom temperatures ranged from 18.86°C to 21.55°C, while maximum difference between temperatures measured before and after scanning was 0.56°C.

**FIGURE 2:**
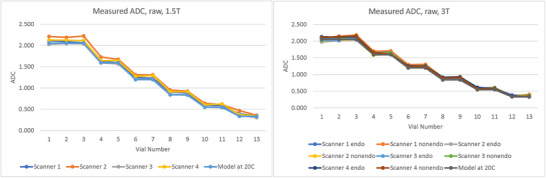
Raw ADC measurements in units of 10^−3^ mm^2^/s of the 13 vials on 1.5 T (left) and 3 T (right) scanners before any normalization was applied.

Raw ADC measurements, without any normalization applied, are presented in Figure 2. While ADC values were centered on discrete values dependent on PVP concentration, some variation was observable in the raw plots. Since ADC varies with PVP concentration, it was necessary to normalize them to reference values to compare relative differences and variation. Mean ADC values across scanners and protocols are listed in Table 2.

**TABLE 2:** Mean raw ADC measurements in units of 10^−3^ mm^2^/s for each vial measured in 1.5 T scanners, 3 T scanners, and protocols, and all scanners and protocols combined.

Vial	1.5 T	3 T	Combined
1 (0%)	2.120	2.056	2.077
2 (0%)	2.112	2.079	2.090
3 (0%)	2.110	2.118	2.115
4 (10%)	1.646	1.634	1.638
5 (10%)	1.620	1.632	1.628
6 (20%)	1.260	1.238	1.245
7 (20%)	1.259	1.254	1.256
8 (30%)	0.905	0.885	0.892
9 (30%)	0.886	0.905	0.898
10 (40%)	0.594	0.574	0.581
11 (40%)	0.589	0.580	0.583
12 (50%)	0.398	0.348	0.365
13 (50%)	0.336	0.357	0.350

**FIGURE 3:**
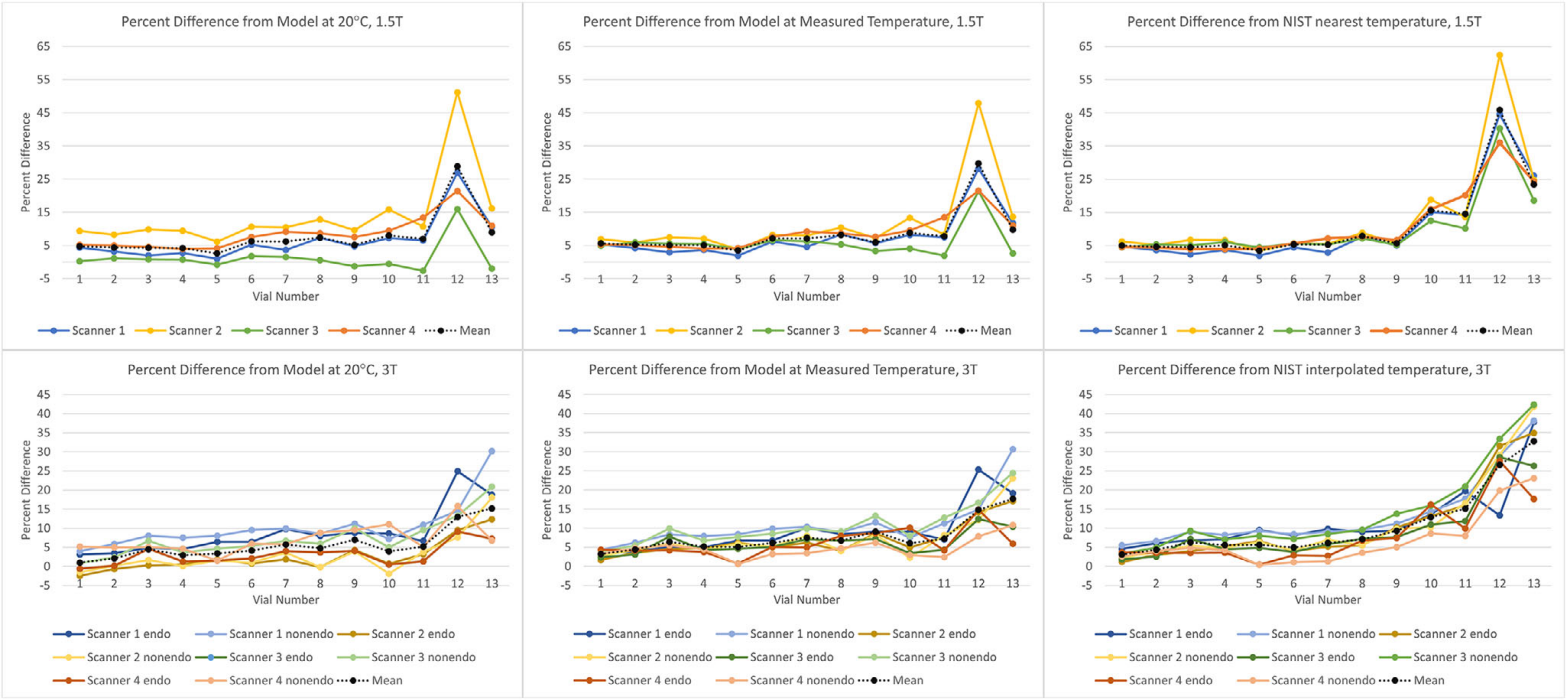
Percent difference of each vial plotted for 1.5 T scanners with the endorectal protocol (top row) and 3 T scanners with both endorectal and non‐endorectal protocols (bottom row). The values were normalized to ADCs_20C_ (left), ADCs_Temp_ (center), and ADCs_NIST_ (right). Limited range and line plots are used to better visualize patterns in individual scanners.

**FIGURE 4:**
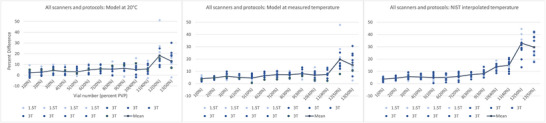
Percent difference of each vial plotted for scanners and protocols. The values were normalized to ADCs_20C_(left), ADCs_Temp_ (center), and ADCs_NIST_ (right).

Figure 3 summarizes the percent difference of each vial for each scanner when the measured ADC values were normalized to the three reference values. Though percent difference generally trended upward with PVP concentration for all three reference values, the values taken from the NIST phantom exhibited the greatest percent difference at high concentrations of 40% and 50%. The use of modeled reference values calculated from the measured temperatures brought the plots together within the tightest range. The percent difference for all scanners is plotted for all three sets of reference values in Figure 4.

**FIGURE 5:**
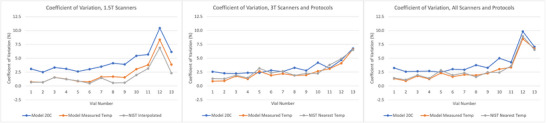
Coefficients of variation between 1.5 T (left), 3 T (middle), and all (right) scanners and protocols for each vial plotted for the three sets of reference values.

The CVs of each vial, when normalized to the three sets of reference values, are illustrated in Figure 5 and listed in Table 3. CV generally trended upward with increasing PVP concentration and decreasing ADC values. Overall, across all vials, scanners, and protocols, using ADCs_Temp_ resulted in the smallest CVs when compared to ADCs_20C_ (*p* = 8.0 × 10^−11^) and ADCs_NIST_ (*p* = 0.019), while using ADCs_20C_ resulted in the greatest CVs. Significant differences in CV for individual vials are also noted in Table 3. With only 4 scanners measured at 1.5 T, no significant differences were found at that field strength. For PVP concentrations from 0% to 40%, the CV of measured ADC across all scanners ranged from 2.5% to 5.0% when normalized to ADCs_20C_, 0.9% to 3.4% when normalized to ADCs_Temp_, and 1.1% to 3.6% when normalized to ADCs_NIST_. While normalized ADC at 50% varied widely on a percent basis, measured ADC values generally trended higher than predicted ADC values with increasing PVP concentrations.

**TABLE 3:** Coefficients of variation when normalized to the three sets of reference values ADCs_20C_, ADCs_Temp_, and ADCs_NIST_.

	1.5 T			3 T			Combined		
Vial	ADCs_20C_	ADCs_Temp_	ADCs_NIST_	ADCs_20C_	ADCs_Temp_	ADCs_NIST_	ADCs_20C_	ADCs_Temp_	ADCs_NIST_
1 (0%)	3.1	0.7	0.7	2.6^**^	0.9	1.3	3.3^**^	1.3	1.4
2 (0%)	2.5	0.7	0.7	2.3^**^	0.9	1.3	2.6^**^	0.9	1.1
3 (0%)	3.3	1.6	1.5	2.2	1.8	1.9	2.7	1.8	2.0^*^
4 (10%)	3.1	1.3	1.2	2.4^*^	1.3	1.5	2.7^*^	1.3	1.4
5 (10%)	2.6	0.9	0.9	2.4	2.6	3.2	2.5	2.3	2.8^*^
6 (20%)	3.0	0.7	0.5	2.8^*^	1.9	2.4	3.0^*^	1.7	1.9
7 (20%)	3.5	1.6	1.5	2.6	2.2	2.6^*^	2.9	2.0	2.4
8 (30%)	4.1	1.7	0.6	3.3^*^	1.9	1.9	3.8^**^	1.9	1.6
9 (30%)	3.9	1.6	0.6	2.8^*^	2.0	2.3	3.3^*^	2.3	2.5
10 (40%)	5.4	3.0	2.0	4.2^*^	2.7	2.3	5.0^**^	3.0	2.4^*^
11 (40%)	5.7	3.8	3.1	3.2	3.1	3.8	4.3	3.4	3.6
12(50%)	10.4	8.3	6.9	4.7	4.1	4.9^*^	9.8	8.5	9.0
13(50%)	6.1	3.9	2.3	6.8	6.6	6.5	7.1	6.7	6.5

^*^ *p* < 0.05 when compared to ADCs_Temp_.

^**^ *p* < 0.002 when compared to ADCs_Temp_.

## 4 DISCUSSION

In this study, we designed a QA procedure to evaluate the reproducibility of our dedicated prostate DWI sequence measured on multiple scanners from a single vendor located at four geographical sites in our institutional network. This procedure incorporated a temperature model that enabled measurement of ADC at room temperature. By using this temperature model as a correction factor in this procedure, we found that ADC, as measured by our prostate diffusion sequences, varied with a maximum CV of 3.4% for all scanners and protocols, for all vials with PVP concentrations up to 40%. The two vials with PVP concentration of 50% exhibited higher CV's of 8.5% and 6.7%, but their mean measured ADCs of 0.350 and 0.346 × 10^−3^ mm^2^/s were well below what is expected for high Gleason grade prostate tumors.^3^, ^5^, ^32^, ^33^, ^34^, ^35^ The smallest CV of 0.9% was measured in vial 2, reflecting 0% PVP and located at the inner ring of the phantom.

Our prostate protocols are designed to adhere to the technical specifications recommended by the Prostate Imaging Reporting and Data System (PI‐RADS).^36^ This QA procedure was designed to be simple while still retaining the ability to evaluate key factors that would affect ADC values measured on a scanner. Since the receive field of an endorectal coil would not provide adequate coverage of the phantom, the phantom was imaged using the spine array coil in the scanner table, which the coil used for posterior signal in prostate imaging. The array coil provided adequate SNR and enabled parallel imaging, obviating the need for an additional torso array coil over the phantom. Parallel imaging is crucial in reducing distortions in EPI‐based DWI as used in this protocol. While prostate imaging is typically performed in an oblique axial plane, positioning the phantom for coronal imaging provided homogeneous signal reception in coronal planes, and the ease of setup in this orientation should also improve reproducibility when measured by multiple users.^20^ By adhering to guidelines provided by PI‐RADS, the scan parameters generally meet ideal and target scan parameters for clinical prostate DWI protocols as provided by the QIBA profile, except that PI‐RADS allows in‐plane pixel dimensions of up to 2.5 mm. PIRADS also allows for generation of synthetic or calculated high *b*‐value images from data acquired with lower *b*‐values, which, combined with the relaxed pixel size requirements, adds more SNR to clinical prostate protocols. If our proposed QA procedure is implemented in practice, reproducibility of the acquired ADC values is ensured if running the same scan parameters produces ADC values in line with baseline ADC values; any measured values outside of expectation would likely be reflective of system deficiencies or deviations. There are several differences between the scan parameters in our protocol and those in the QIBA DWI phantom scan procedure, which may result in differences when comparing measurements. Notably, the QIBA scan procedure acquires with phantom temperature fixed at 0°C, scans in the axial plane in a head coil, uses higher in‐plane resolution, avoids phase oversampling, and uses *b*‐values of 0, 500, 900, and 2000 s/mm^2^, compared to *b*‐values of 50 and 800 s/mm^2^ in our protocol. The greater number of b‐values would improve fitting for a greater range of ADC values, but the higher maximum *b*‐value also increases the minimum achievable TE and decreases SNR.

To our knowledge, this study is the first use of temperature correction with a PVP phantom for QA of a clinical protocol at room temperature. There are some advantages to measuring ADC of the NIST phantom at room temperature over measuring at 0°C. Since ADC increases with increasing temperature, more of the vials would have ADCs in physiological range. There are also less susceptibility‐induced distortions in the images, which may be caused by ice in the phantom water.^19^ Overall, the procedure requires less time to prepare, which was the primary motivation of this study. However, since temperature of the phantom is not tightly controlled, some care must be taken in maintaining and accurately measuring the temperature. Scanner room temperature is controlled by central air conditioning and heating systems and generally varies with a range of 2°C–3°C. On the other hand, no controls are typically placed on the temperature of cold tap water. This potentially introduces considerable variation to the phantom temperature, but the water can be easily adjusted with the addition of hot tap water or ice. Since the temperature cannot be controlled or measured once the phantom is in the scanner, a rough adjustment to room temperature reduces potential temperature changes in the phantom during scanning, and measurement of the temperature before and after scanning enables a more precise estimate of the temperature of the phantom during the DWI sequence. In our study, the thermometer used to measure the phantom water was the same one that was used to measure air temperature in the scanner room. Measurement of air is not as stable as that of water, and care must be taken to ensure the thermometer is dry and away from air currents. Even so, air temperature measured by this method fluctuated by a few tenths of a degree when observed over the course of a minute. A rough mean estimate of air temperature was subjectively recorded, but this potential error in measured room temperature is still well below our target of adjusting phantom water temperature to within ± 0.5°C. Recently, a novel phantom design was introduced, which can provide a measure of temperature at the time of imaging using an embedded thermometer that can be read directly from the MR image.^27^, ^37^ Our study demonstrates the improvements in reproducibility that can be achieved when correction based on measured temperature is used in lieu of strict temperature control.

Most multisite studies have used distilled water at 0°C or have focused analysis on vial 1 of the NIST phantom, which is a vial of pure distilled water at the center of the phantom and serves as the standard of measurement for many previous studies, as well as conformance to the QIBA profile. When corrected with the temperature model, our measured CV of 1.3% in vial 1 compares favorably to other multi‐scanner and multisite studies, which range from 1.1% to 2.8%.^10^, ^16^, ^38^, ^39^ While not specified for multi‐scanner analysis, the maximum CV recommended by QIBA profile for long‐term repeatability of a single scanner is 2.2%. The scanners in our study were all manufactured by the same vendor and split evenly between the two field strengths, though the model of one 3 T scanner was distinct from the other three. While CVs for vial 1 were even tighter within field strengths, the scheduling system at our clinic prioritizes patient and clinical resource availability and does not tightly restrict which scanner a patient is assigned to, thus necessitating evaluation across all scanners to be meaningful in practice.

The percent difference calculation used in our study is mathematically identical to the percent bias calculation used to characterize the difference between a measured value and a reference value.^7^ In our study, we did not take any of the reference values as a true gold standard, and hence the percent difference calculation would not be accurately described as a bias calculation. However, our clinical goal was to reduce or eliminate the effect of temperature as a confounding factor when measuring reproducibility of our prostate ADC measurements across scanners. Because of this, no gold standard ADC measurement was performed on our specific phantom at each phantom temperature. For the highest PVP concentration of 50% and a temperature of 20°C, the ADC given by the temperature model was roughly similar to the NIST measurement (8.5% and 6.7% vs. 9.0% and 6.5%), while other concentrations in the range of 0% to 30% differed by up to 2.77%, which may reflect slight differences in the solutions and the measurement methods. The ADC measurements in our study were generally in better agreement with the temperature model fixed at 20°C than measured values from NIST. Through personal communication with CalibreMRI, the published NIST measurements at room temperatures were performed on a recent batch made with an updated manufacturing process. The vial solutions have also been observed to deviate over time, which could impact our phantom manufactured over 4 years prior to the study. Since our measurements trended higher than the NIST measurements, it may also be possible that any motion or vibration may have contributed to the apparent diffusion in our measurements, especially at the higher PVP concentrations and lower diffusivity values.

There were limitations to this study. Since we did not perform repeat measurements for all scanners and all protocols, only one measurement per scanner and protocol was included in this analysis. Any repeat measurements performed on individual scanners or during trial runs of the QA procedure were excluded. This results in a broad sampling across scanners and protocols, but limited numbers for any given field strength or protocol, and insufficient data to assess intra‐scanner repeatability of all scanners. Hence, one would not be able to separate out the effects of intra‐scanner repeatability on the reproducibility of the scanner network as a whole. This data also could not be used to demonstrate the efficacy or utility of temperature correction on individual DWI measurements, if such corrections were to be implemented clinically on patients. Assessment of *b*‐value dependence also would not be possible due to the single high *b*‐value of 800 s/mm^2^. This value used in our prostate protocol was not ideal for measurement of the highest concentration vials (12 and 13), which demonstrated the greatest variability and would require higher *b*‐values as used in the QIBA profile to improve precision.^16^ A sample with ADC of 0.35 × 10^−3^ mm^2^/s would lose only about 23% of its signal between applied *b*‐values of 50 and 800 s/mm^2^, and small signal differences in this ADC range would result in proportionally larger differences in fitted ADC. The ADC values in these vials are also much lower than ADC values typically expected of prostate tumors.^40^

## 5 CONCLUSION

ADC variance due to temperature can be reduced by applying a model for predicted ADC that takes temperature into account. By reducing or eliminating temperature as a factor, observed variances of ADC values can more accurately be attributed to other factors, enabling more effective troubleshooting as needed. This approach permits a simplified QA process for ADC measurement when employing a protocol across multiple scanners at multiple, separate geographic sites.

## Author Contributions

**Ken‐Pin Hwang**: Conceptualization (equal); methodology (lead); investigation (lead); formal analysis (lead); writing—original draft (lead); writing—review and editing (equal). **Joshua Yung**: Conceptualization (equal); methodology (supporting); writing—review and editing (equal). **R. Jason Stafford**: Conceptualization (equal); methodology (supporting); writing—review and editing (equal); supervision (supporting). **Caroline Chung**: Conceptualization (equal); funding acquisition (lead); supervision (supporting). **Aradhana M. Venkatesan**: Conceptualization (equal); writing—review and editing (equal); supervision (lead).

## CONFLICT OF INTEREST STATEMENT

This work was supported by Siemens Healthineers and by the University of Texas MD Anderson Cancer Center‐Siemens Alliance. Ken‐Pin Hwang has received research support from GE Healthcare and Siemens Healthineers. R. Jason Stafford, Caroline Chung, and Aradhana Venkatesan have also received research support from Siemens Healthineers.
